# The combined internal and external fixation surgery is effective and safe in treating posterior lateral tibial plateau fractures: An observational study

**DOI:** 10.1097/MD.0000000000038572

**Published:** 2024-09-06

**Authors:** Yuechao Guo, Wen An, Jialiang Ma, Zhe Wang, Yujian Zhang

**Affiliations:** a Orthopedics Department, The First Hospital of Qinhuangdao, Qinhuangdao, Hebei, China.

**Keywords:** internal and external combined plate fixation, knee joint function, quality of life, tibial plateau fracture

## Abstract

To investigate the treatment outcomes of combined internal and external fixation surgery for patients with posterior lateral tibial plateau fractures and explore its safety. The study was conducted from February 2020 to February 2023 and included a total of 77 patients with Schatzker IV and Schatzker V type posterior lateral tibial plateau fractures. Patients were divided into control group and treatment group according to different treatment methods: the control group with 38 cases received treatment with dual-support plates, and the study group with 39 cases received treatment with internal fixation using medial plates combined with lateral locking plates. Clinical indicators during treatment, immediate postoperative and 12-month postoperative radiographic indicators, Rasmussen knee joint function scores before and 3 months after surgery, knee joint function recovery, quality of life, and postoperative complications were recorded and compared between the 2 groups. The inter-group comparisons were made for intraoperative blood loss, surgical duration, and the time to start weight-bearing postoperatively (*P* > .05). The study group had shorter postoperative hospital stays and fracture healing times compared to the control group (*P* < .05). Immediately postoperatively, the medial tilt angle and posterior tilt angle in both groups were compared (*P* > .05). At 12 months postoperatively, the medial tilt angle decreased and the posterior tilt angle increased in both groups compared to immediately postoperative values (*P* < .05), with no significant difference between the groups (*P* > .05). However, at 3 months postoperatively, the scores for various dimensions in both groups increased compared to preoperative values, and the study group had higher scores than the control group (*P* < .05). However, at 3 months postoperatively, the quality of life scores were higher than preoperative values in both groups, with the study group having higher scores (*P* < .05). The occurrence of complications during the treatment period was compared between the 2 groups (*P* > .05). The medial and lateral combined plate fixation has a good clinical effect in the treatment of posterolateral tibial plateau fractures, which can shorten the fracture healing time, help the recovery of knee joint function and improve the quality of life of patients after operation, and has high safety in the treatment process.

## 1. Introduction

Schatzker type IV tibial plateau fractures occur on the inner side of the tibial plateau and often involve the posterior lateral aspect. Schatzker type V tibial plateau fractures are bilateral plateau fractures, often resulting from high-energy trauma such as car accidents or falls.^[1]^ In most cases, complex tibial plateau fractures are often associated with damage to the soft tissues, nerves, and blood vessels around the knee joint, and sometimes they may even involve dislocation of the knee joint. The tibial plateau is a critical bone structure in the human body, supporting the bending and extension functions of the knee joint.^[2]^ As a major weight-bearing joint, when a partial tibial plateau fracture occurs in the knee joint, it severely limits its function, causing inconvenience in daily life, and may even lead to lower limb disability, having a profound impact on the patient’s physical and psychological well-being.^[3]^ When dealing with complex tibial plateau fractures, the key lies in accurately repositioning the damaged joint and providing stable internal fixation. This not only effectively corrects the alignment of the lower limb but also restores the integrity of the joint surface. Therefore, such surgeries not only require doctors to possess a high level of surgical skills but also demand the correct selection of appropriate internal fixation materials. Although traditional bilateral support plate fixation surgery provides good results in stabilizing Schatzker IV and Schatzker V tibial plateau fractures, during bilateral support plate fixation, the periosteum at the fracture site experiences additional pressure. This excessive compression may not only lead to periosteal damage but also affect the blood supply to the fracture site, thereby delaying the healing process of the fracture.^[4]^ Compared to the limitations of traditional double-support steel plate treatment, locked plate fixation has shown significant advantages in the treatment of complex tibial plateau fractures. These advantages include providing stable fixation, employing minimally invasive techniques, and featuring point contact, all of which contribute to maintaining the stability of the biomechanical structure. This ensures solid fixation of the fracture site and ensures good blood supply, thereby promoting rapid fracture healing. Especially for patients with comminuted and extra-articular tibial plateau fractures, locked plate fixation has become the preferred method for treating such fractures.^[5]^ In light of this, the aim of this study is to analyze the therapeutic effects of internal and external dual-plate fixation surgery on patients with posterior lateral tibial plateau fractures and investigate its safety. It is hoped that this study will provide empirical evidence for future research on the treatment methods of complex tibial plateau fractures.

## 2. Materials and methods

### 2.1. General data

This study was conducted from February 2022 to February 2023 and included a total of 77 patients with Schatzker IV and V type fractures of the posterior lateral tibial plateau, as well as Schatzker V type tibial plateau fractures. According to different treatment methods, the 2 groups of patients were divided into control group and experimental group: a control group consisting of 38 patients who received treatment with dual-support steel plates, and a study group consisting of 39 patients who received treatment with internal fixation using medial steel plates in combination with lateral locking steel plate fixation. The general data between the 2 groups were compared (*P* > .05), see Table 1. This study was approved by the hospital ethics committee. Schatzker classification as follows: Schatzker I: wedge-shaped pure cleavage fracture of the lateral tibial plateau, originally defined as having <4 mm of depression or displacement. Schatzker II: splitting and depression of the lateral tibial plateau; namely, type I fracture with a depressed component (generally considered commonest 5). Schatzker III: pure depression of the lateral tibial plateau; divided into 2 subtypes. Schatzker IV: medial tibial plateau fracture with a split or depressed component. Schatzker V: wedge fracture of both lateral and medial tibial plateau. Schatzker VI: transverse tibial metadiaphyseal fracture, along with any type of tibial plateau fracture (metaphyseal–diaphyseal discontinuity).

**Table 1:** Comparison of general data between the 2 groups.

Group	Gender		Age (yrs old)	Time from fracture to admission (h)	Causes of injury			
	Male	Female			Traffic trauma	High falling injury	Fall injury	Bruise
Control group (n = 38)	22 (57.89)	16 (42.11)	44.12 ± 8.79	4.22 ± 1.36	15 (39.47)	13 (34.21)	5 (13.16)	5 (13.16)
Study group (n = 39)	21 (53.85)	18 (46.15)	44.98 ± 9.15	4.12 ± 1.55	12 (30.77)	11 (28.21)	9 (23.06)	7 (17.95)
χ^2^/*t*	0.128		−0.420	0.301	1.964			
*P*	.721		.675	.765	.580			

### 2.2. Inclusion and exclusion criteria

*Inclusion criteria:* The patient was confirmed as Schatzker type IV and V fractures of the tibial plateau by clinical CT; patients with a clear history of trauma, fracture injury for fresh closed; patients were unilateral fractures; no previous related knee joint disease; patients with good compliance, can follow the doctor’s advice for rehabilitation exercise and follow-up.

*Exclusion criteria:* There are old fractures; patients with severe medical diseases, or preoperative assessment, there is a serious malnutrition, cannot tolerate surgery; there were multiple fractures and multiple traumas; the clinical data of the patients were incomplete.

### 2.3. Methods

The study group underwent treatment with internal fixation using medial steel plates in combination with external fixation using lateral locking steel plates: The surgery is performed under continuous epidural anesthesia, with the patient in a supine position. Hemostasis is achieved by using a tourniquet at the base of the thigh while maintaining pressure control at 78.9 kPa. During the surgery, the injured limb is positioned in abduction. The incision is made along the posterior inner edge of the proximal tibia and tissues are separated parallel to it to facilitate the surgical procedure. Subsequently, the “goose’s foot” structure is exposed, and by applying traction to both sides, the fracture area of the proximal tibia’s inner shaft is revealed. During the surgical procedure, the affected limb is appropriately elevated. The lateral incision is made along the axis of the knee joint, with a distance of at least 7 cm from the skin incision on the inner side, starting at Gerdy tubercle and curving it, then continuing along this path to reach approximately 1 cm on the front outer edge of the tibia. The fascia is incised below the meniscus to adequately expose the lateral tibial plateau area and the joint surface. First, the joint surface and the fractured tibial shaft are repositioned. Autologous iliac bone graft is used to fill any bone defects beneath the joint surface. Then, fixation is achieved using an external locking steel plate and an internal supporting steel plate to complete the reduction procedure. An anatomical titanium plate is installed on the medial side of the tibia. This is done by drilling holes first, followed by tapping and threading, and then inserting screws to secure it in place. The procedure begins by using cortical bone screws for compression fixation of the support fracture fragments. Subsequently, locking or cortical bone screws are used to maintain compression of the steel plate. The lateral steel plate is inserted using a navigational frame through an approach between the anterior tibial muscle and the periosteum. Initially, the locking steel plate is fixed on the lateral side. Then, a guide pin is inserted through the proximal end of the plate using a Kirschner wire, and the plate’s position is adjusted with the assistance of a C-arm X-ray machine. Finally, drilling and probing are completed using a guide, and locking screws are used for fixation.

**Figure 1.**
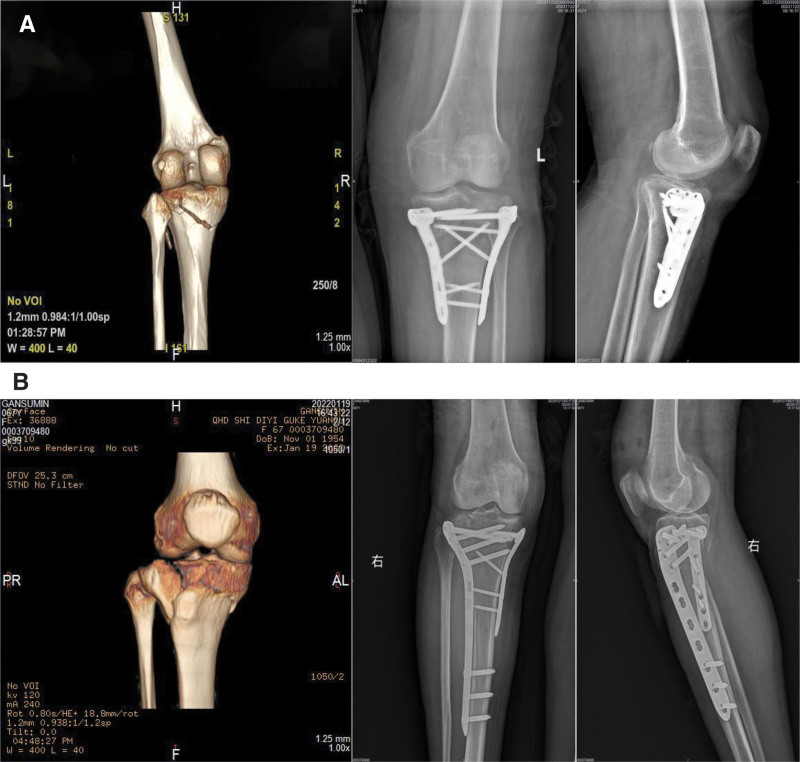
Figure (A) shows the preoperative and postoperative X-ray images of a patient with a tibial plateau fracture treated with compression fixation, and (B) shows the preoperative and postoperative X-ray images of another patient with a tibial plateau fracture treated with compression fixation.

The control group received treatment with dual-support steel plates: aside from using dual-support steel plates as the fixation material, the fixation method was the same as that in the study group. After achieving the desired fracture reduction and fixation, wound irrigation and suturing were performed. Subsequently, a negative pressure drainage tube was placed, and dressing was applied (Fig. 1).

### 2.4. Observed indexes

During the treatment of the patients, various clinical indicators were recorded, including surgical time, blood loss, fracture healing time, length of hospital stay, and the time to begin weight-bearing postoperatively. Additionally, the radiological indicators at immediate post-operation and 12 months post-operation were documented, which included measurements of the medial tilt angle and posterior slope angle. Furthermore, assessments of knee joint function in patients were conducted using the Rasmussen knee joint function scoring system at both preoperative and 3 months postoperative time points. The patients’ quality of life was also evaluated using the SF-36 health survey questionnaire. Any occurrence of complications was documented as well.

The Rasmussen Knee Joint Function Score^[6]^ is a comprehensive assessment of 4 aspects in patients: pain level, range of motion, walking ability, and stability. A higher score indicates better recovery of knee joint function in patients.

The SF-36 Health Survey Short-Form^[7]^ is used to assess various aspects of a patient’s health-related quality of life. It evaluates physical functioning, physical role, bodily pain, general health, vitality, social functioning, emotional role, and mental health. A higher score on the SF-36 indicates a higher quality of life for the patient.

### 2.5. Statistical processing

Statistical analysis was performed using SPSS26.0 software. The count data was expressed as n (%), and the line 2 test was performed. The measurement data were expressed as ($\bar{\chi }\pm s$), and the independent sample *t* test was performed. The significance level was set to *P* < .05.

## 3. Results

### 3.1. Comparison of clinical indicators

The comparison was made between the 2 groups in terms of surgical time, intraoperative blood loss, and time to initiate weight-bearing (*P* > .05). However, the study group had significantly shorter postoperative hospital stays and faster fracture healing times compared to the control group (*P* < .05). See Table 2.

**Table 2:** Comparison of clinical indicators.

Group	Operation time (min)	Intraoperative blood loss (mL)	Hospital stay after operation (d)	Fracture healing time (mo)	The time of weight-bearing after operation (d)
Control group (n = 38)	95.44 ± 8.97	277.21 ± 19.26	8.54 ± 2.65*	3.67 ± 1.38	44.06 ± 5.11
Study group (n = 39)	94.98 ± 9.45	276.26 ± 15.59	6.11 ± 2.12*	3.02 ± 1.31	43.15 ± 6.89
*t*	0.219	0.238	4.449	2.120	0.657
*P*	.827	.812	<.001	.037	.513

* Indicates *P* < .05.

### 3.2. Comparison of imaging indicators

Immediately after the surgery, the medial tilt angle and posterior slope angle were compared between the 2 groups (*P* > .05). However, at the 12-month postoperative mark, both groups showed a decrease in the medial tilt angle and an increase in the posterior slope angle compared to immediately after the surgery (*P* < .05). Nevertheless, when comparing the 2 groups, there were no significant differences (*P* > .05). See Table 3.

**Table 3:** Comparison of imaging indicators.

Group	In-over angle (°)		Posterior tilt angle (°)	
	Immediate postoperative	12 mo after operation	Immediate postoperative	12 mo after operation
Control group (n = 38)	85.55 ± 6.15	81.23 ± 4.15*	4.05 ± 0.64	4.61 ± 0.74*
Study group (n = 39)	85.12 ± 5.49	82.44 ± 4.23*	4.01 ± 0.59	4.58 ± 0.61*
*t*	0.324	−1.267	0.285	0.194
*P*	.747	.209	.776	.846

* Compared with the same group immediately after operation, *P* < .05.

### 3.3. Comparison of Rasmussen knee function scores

Before the surgery, the pain level, range of motion, walking ability, and stability were compared between the 2 groups (*P* > .05). However, at the 3-month postoperative mark, both groups showed increased scores in each dimension compared to preoperative scores, and the study group had higher scores than the control group (*P* < .05). See Table 4

**Table 4:** Comparison of Rasmussen knee function scores.

Group	Severity of pain (score)		Range of motion (score)	
	Before operation	3 mo after operation	Before operation	3 mo after operation
Control group (n = 38)	18.11 ± 3.46	20.55 ± 3.56*	19.03 ± 2.15	20.65 ± 2.15*
Study group (n = 39)	17.99 ± 2.56	23.57 ± 2.98*	19.11 ± 1.98	23.08 ± 2.78*
*t*	0.173	−4.041	−0.170	−4.256
*P*	.863	<.001	.866	<.001
Group	Walking ability (score)		Stability (score)	
	Before operation	3 mo after operation	Before operation	3 mo after operation
Control group (n = 38)	17.81 ± 2.13	20.55 ± 2.15*	19.23 ± 3.05	21.55 ± 2.05*
Study group (n = 39)	17.45 ± 2.98	24.58 ± 1.96*	19.33 ± 3.46	23.66 ± 3.11*
*t*	0.608	−8.600	−0.134	−3.505
*P*	.545	<.001	.893	.001

* Compared with the same group before operation, *P* < .05.

### 3.4. Comparison of quality of life scores

Before the surgery, the various aspects of quality of life scores were compared between the 2 groups (*P* > .05). However, at the 3-month postoperative assessment, both groups showed increased scores in all dimensions compared to preoperative scores, with the study group having higher scores (*P* < .05). See Table 5

**Table 5:** Comparison of quality of life scores.

Group	Physical function (score)		Physical role (score)	
	Before operation	3 mo after operation	Before operation	3 mo after operation
Control group (n = 38)	43.15 ± 8.46	61.33 ± 7.89*	48.66 ± 8.16	61.29 ± 9.79*
Study group (n = 39)	43.56 ± 7.59	68.98 ± 6.49*	49.11 ± 9.17	69.65 ± 9.15*
*t*	−0.224	−4.652	−0.227	−3.872
*P*	.823	<.001	.821	<.001
Group	Body pain (score)		General health (score)	
	Before operation	3 mo after operation	Before operation	3 mo after operation
Control group (n = 38)	40.98 ± 9.12	58.79 ± 9.67*	45.44 ± 7.44	63.98 ± 8.78*
Study group (n = 39)	41.22 ± 10.12	69.98 ± 8.78	45.12 ± 6.59	69.98 ± 7.15*
*t*	−0.109	−5.319	0.200	−3.292
*P*	.913	<.001	.842	.002
Group	Vitality (score)		Social function (score)	
	Before operation	3 mo after operation	Before operation	3 mo after operation
Control group (n = 38)	45.44 ± 8.97	72.45 ± 9.64*	56.16 ± 9.41	76.56 ± 6.45*
Study group (n = 39)	45.19 ± 7.26	79.65 ± 8.49*	55.98 ± 8.49	79.98 ± 8.79*
*t*	0.135	−3.480	0.088	−2.382
*P*	.893	.001	.930	.020
Group	Emotional role (score)		Mental hygiene (score)	
	Before operation	3 mo after operation	Before operation	3 mo after operation
Control group (n = 38)	53.26 ± 8.79	72.87 ± 8.58*	65.11 ± 9.46	73.68 ± 6.98*
Study group (n = 39)	52.87 ± 7.12	76.92 ± 8.15*	64.99 ± 5.48	76.88 ± 5.98*
*t*	0.214	−2.124	0.068	−2.162
*P*	.831	.037	.946	.034

* Compared with the same group before operation, *P* < .05.

### 3.5. Occurrence of complications

The occurrence of complications during the treatment period was compared between the 2 groups (*P* > .05). See Table 6

**Table 6:** Occurrence of complications (%).

Group	Wound infections	Delayed fracture healing	Ankylosis	Reduction loss	Total occurrence
Control group (n = 38)	1 (2.63)	2 (5.26)	3 (7.89)	2 (5.26)	8 (21.05)
Study group (n = 39)	0 (0.00)	1 (2.56)	1 (2.56)	2 (5.13)	4 (10.26)
χ^2^					1.705
*P*					.192

## 4. Discussion

Schatzker IV type tibial plateau fracture is characterized by involvement of the posterior lateral portion of the tibia,^[8]^ whereas Schatzker V type plateau fracture represents bilateral plateau fractures. When the fracture extends to involve the tibial plateau, the primary treatment goal is to restore the normal biomechanical axis of the lower limb through precise anatomical reduction and to ensure stable fixation of the joint surface to correct any deformities.^[9,10]^

The key focus is on achieving the correct alignment and smoothness of the joint while initiating early functional rehabilitation training to accelerate the healing process of the fracture. The goal is to maximize the restoration of normal knee joint function.^[11,12]^ This study evaluated the clinical indicators of 2 surgical methods and found that there were no significant differences between the 2 groups in terms of surgical duration, intraoperative blood loss, and the time to initiate weight-bearing postoperatively. This suggests that the combined internal and external fixation method is comparable to the traditional approach in terms of surgical time and trauma. It also indicates the feasibility and safety of the surgical technique used in the study group. In contrast, the study group had significantly shorter postoperative hospital stays and faster fracture healing times compared to the control group. This implies that the combined internal and external fixation surgery accelerated the healing process of the fracture and reduced the overall recovery time for patients. Additionally, in terms of limb function recovery, data at the immediate postoperative and 12-month follow-up showed no significant differences between the 2 groups in terms of medial tilt angle and posterior slope angle. This indicates that both surgical methods were equally effective in maintaining the anatomical structure of the knee joint. Furthermore, when comparing Rasmussen Knee Joint Function scores before and 3 months after the surgery, both groups of patients showed improvements in their scores, but the study group demonstrated more significant improvements. This indicates that the combined internal and external fixation surgery is more favorable for enhancing knee joint function and overall recovery in patients.

The reason for the observed differences may be attributed to the fact that while the use of dual-support steel plates provides stable fixation, this method can potentially subject the periosteum in the fracture area to higher pressure. In some cases, it may even lead to compressive injuries. This excessive pressure can interfere with the blood supply to the fracture site, increase the risk of postoperative complications, and potentially affect the effective reduction of the fracture.^[13,14]^ Based on the factors mentioned above, patients undergoing early functional exercises after surgery are often limited in their ability, which can result in less than ideal knee joint recovery. However, in the method of using the lateral locking plate and medial support plate fixation, by maintaining an angle offset of approximately 5° between the locking screws and the plates, this technique reduces pressure on the periosteum, thereby avoiding ischemic damage to the periosteum and not interfering with the blood supply to the fracture site.^[15]^ Moreover, the use of locking screw fixation ensures a strong and stable fixation of the fracture site, maintaining good biomechanical performance of the fracture ends and reducing the risk of loosening or detachment. This method not only provides effective shear force fixation but also supports and maintains the stability of the joint surface, preventing fracture displacement.^[16,17]^ Therefore, this approach ensures that patients can initiate early postoperative functional rehabilitation exercises, which is beneficial for both rapid fracture healing and promoting knee joint function recovery.

The results of this study also indicated that there were no differences in various aspects of quality of life scores between the groups before the surgery. However, at the 3-month postoperative assessment, both groups showed increased scores in all dimensions compared to preoperative scores, with the study group having higher scores. This further reflects the advantages of combined internal and external fixation surgery in improving the quality of life for patients. Furthermore, there were no significant differences in the occurrence of complications between the 2 groups, indicating that the surgical technique used in the study group is as safe as the traditional method. This is because the unique properties of locking plates effectively reduce damage to the periosteum while ensuring good blood flow at the fracture site, which contributes to accelerating fracture repair.^[18]^ Moreover, this method provides both efficient and stable fixation, helping to maintain the smoothness of the joint surface. As a result, patients can start their recovery training earlier and, therefore, recover normal knee joint function faster, improving their quality of life.^[19,20]^

This study still has some limitations: the sample size is too small; this study is a retrospective study; the follow-up time is short, and the long-term prognosis of patients cannot be observed. Therefore, in the future plan, we plan to conduct a multi-center RCT study and increase the follow-up time to observe the long-term prognosis of patients. In order to find a better treatment for posterior lateral fracture of tibial plateau.

In summary, the combined internal and external fixation surgery for treating lateral tibial plateau fractures with associated Schatzker IV and V types demonstrates favorable clinical outcomes. It can reduce fracture healing time, enhance postoperative functional recovery, improve the quality of life for patients, and ensure good safety during the treatment process.

## Author contributions

**Conceptualization:** Yuechao Guo, Wen An, Jialiang Ma, Zhe Wang, Yujian Zhang.

**Data curation:** Yuechao Guo, Wen An, Jialiang Ma, Zhe Wang, Yujian Zhang.

**Formal analysis:** Yuechao Guo, Wen An, Jialiang Ma, Zhe Wang, Yujian Zhang.

**Investigation:** Yuechao Guo, Jialiang Ma, Zhe Wang, Yujian Zhang.

**Methodology:** Yuechao Guo, Wen An, Jialiang Ma, Zhe Wang, Yujian Zhang.

**Writing – original draft:** Yuechao Guo, Wen An, Yujian Zhang.

**Writing – review & editing:** Yuechao Guo, Wen An, Zhe Wang, Yujian Zhang.

**Supervision:** Wen An, Jialiang Ma.

**Validation:** Wen An.

**Funding acquisition:** Jialiang Ma.
